# Bioactive Compounds and Biological Functions of Garlic (*Allium sativum* L.)

**DOI:** 10.3390/foods8070246

**Published:** 2019-07-05

**Authors:** Ao Shang, Shi-Yu Cao, Xiao-Yu Xu, Ren-You Gan, Guo-Yi Tang, Harold Corke, Vuyo Mavumengwana, Hua-Bin Li

**Affiliations:** 1 Guangdong Provincial Key Laboratory of Food, Nutrition and Health, Department of Nutrition, School of Public Health, Sun Yat-sen University, Guangzhou 510080, China; 2 Department of Food Science & Technology, School of Agriculture and Biology, Shanghai Jiao Tong University, Shanghai 200240, China; 3 Institute of Urban Agriculture, Chinese Academy of Agricultural Sciences, Chengdu 610213, China; 4 DST/NRF Centre of Excellence for Biomedical Tuberculosis Research, US/SAMRC Centre for Tuberculosis Research, Division of Molecular Biology and Human Genetics, Department of Biomedical Sciences, Faculty of Medicine and Health Sciences, Stellenbosch University, Cape Town 8000, South Africa

**Keywords:** garlic, phytochemicals, organic sulfides, health benefits, antioxidant, anticancer, cardiovascular protection, antimicrobial

## Abstract

Garlic (*Allium sativum* L.) is a widely consumed spice in the world. Garlic contains diverse bioactive compounds, such as allicin, alliin, diallyl sulfide, diallyl disulfide, diallyl trisulfide, ajoene, and S-allyl-cysteine. Substantial studies have shown that garlic and its bioactive constituents exhibit antioxidant, anti-inflammatory, antibacterial, antifungal, immunomodulatory, cardiovascular protective, anticancer, hepatoprotective, digestive system protective, anti-diabetic, anti-obesity, neuroprotective, and renal protective properties. In this review, the main bioactive compounds and important biological functions of garlic are summarized, highlighting and discussing the relevant mechanisms of actions. Overall, garlic is an excellent natural source of bioactive sulfur-containing compounds and has promising applications in the development of functional foods or nutraceuticals for the prevention and management of certain diseases.

## 1. Introduction

Garlic (*Allium sativum* L.) is a common spice with many health benefits, mainly due to its diverse bioactive compounds, such as organic sulfides, saponins, phenolic compounds, and polysaccharides [1,2,3]. Garlic is commonly consumed and has a long history of being utilized as a traditional medicine in China [4]. In recent decades, numerous studies have demonstrated the remarkable biological functions of garlic, including antioxidant, cardiovascular protective, anticancer, anti-inflammatory, immunomodulatory, anti-diabetic, anti-obesity, and antibacterial properties [5,6,7,8,9,10,11]. Investigations have increasingly focused on black garlic, a processed garlic product with increased polyphenol and flavonoid contents, as well as better antioxidant properties, compared to the fresh garlic [12]. In order to highlight the significance of garlic in human health, we searched high-quality studies from the last five years from the Web of Science Core Collection and reviewed the main bioactive compounds and biological functions of garlic, with special attention paid to the relevant mechanisms of actions. We hope that this review paper will attract more interest in garlic and provide updated scientific evidence for the better utilization of garlic in human health and disease management.

## 2. Bioactive Compounds of Garlic 

**Figure 1:**
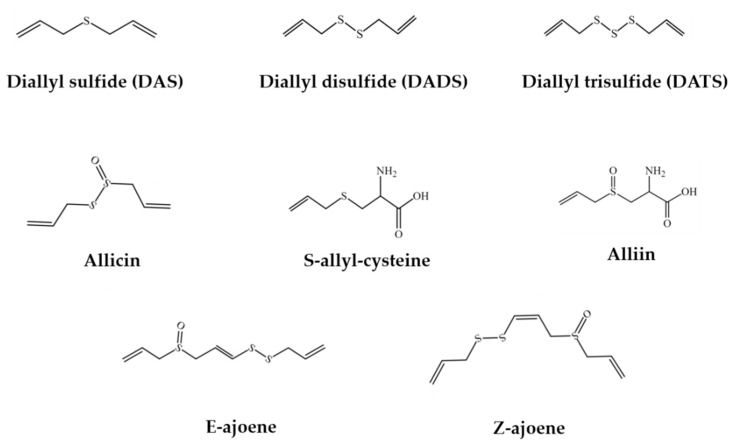
The chemical structures of the main organosulfur compounds in garlic.

Garlic has a variety of bioactive compounds, including organosulfur compounds, saponins, phenolic compounds, and polysaccharides [2,3,13,14]. The major active components of garlic (Figure 1) are its organosulfur compounds, such as diallyl thiosulfonate (allicin), diallyl sulfide (DAS), diallyl disulfide (DADS), diallyl trisulfide (DATS), E/Z-ajoene, S-allyl-cysteine (SAC), and S-allyl-cysteine sulfoxide (alliin) [15,16,17,18]. In general, organosulfur compounds in raw garlic have higher digestibility than those in cooked garlic [19]. In addition, saponins were found to be more stable in the cooking process [20]. The total amount of saponin in purple garlic was almost 40 times higher than that in white garlic, and several saponin compounds were only found to exist in purple garlic, such as desgalactotigonin-rhamnose, proto-desgalactotigonin, proto-desgalactotigonin- rhamnose, voghieroside D1, sativoside B1-rhamnose, and sativoside R1 [2]. Moreover, garlic contained more than 20 phenolic compounds, with higher contents than many common vegetables [21]. The main phenolic compound was β-resorcylic acid, followed by pyrogallol, gallic acid, rutin, protocatechuic acid, as well as quercetin [22]. Furthermore, garlic polysaccharides were reported to contain 85% fructose, 14% glucose, and 1% galactose [23]. 

The effects of different processing methods on bioactive components of garlic have been also studied. For example, it was found that the 38 components of garlic changed after thermal treatment during the processing of black garlic [24]. In addition, the polysaccharide degraded and the content of reducing sugar increased during the thermal processing of black garlic [25]. Also, increasing temperature and decreasing humidity enhance the contents of polyphenols and the total flavonoids in black garlic [26,27]. In the future, more bioactive compounds produced in the processing of garlic should be separated and identified.

## 3. Biological functions of Garlic

### 3.1. Antioxidant Activity

The antioxidant activities of natural products have been widely evaluated, such as fruits, vegetables, mushrooms, cereal, flowers, and wild fruits [28,29,30,31,32,33,34]. Accumulating studies have found that garlic has strong antioxidant properties. A study evaluated the antioxidant capacities of both raw and cooked garlic, and found that the raw garlic exhibited stronger antioxidant activity (by 1,1-diphenyl-2-picrilhydrazyl (DPPH) radical scavenging assay, 2,2’-Azino-bis(3-ethyl- benzothiazoline-6-sulfonic acid) (ABTS) radical scavenging assay, and ferric ion reducing antioxidant power (FRAP) assay). Stir-fried garlic was also shown to have stronger antioxidant capacities (by β-carotene bleaching), indicating that the processing could affect the antioxidant property of garlic [35]. In another study, the results of DPPH and oxygen radical absorption capacity (ORAC) assays showed that the ethanolic extract of garlic sprouts exhibited stronger antioxidant activities than the ethanolic extract of raw garlic [36]. In addition, the antioxidant properties of aged garlic were found to be higher than fresh garlic by DPPH, ABTS, FRAP, H_2_O_2_ scavenging, and Fe^2+^ chelating assays [37]. Compared with multi clove garlic extract, single clove garlic extract had a higher amount of phenolic compounds and showed stronger antioxidant activity [38]. Moreover, the antioxidant activity of black garlic increased with thermal treatment, and the highest antioxidant activity was obtained on the 21st day of processing [39,40]. Also, the increased pressure improved the antioxidant activity of garlic paste [41]. However, the antioxidant activity of “Laba” garlic, a traditional Chinese garlic product, decreased during fermentation [42]. 

The aged garlic extract (AGE) induced the expression of several antioxidant enzymes, such as heme oxygenase-1 (HO-1) and the glutamate-cysteine ligase modifier (GCLM) subunit through the nuclear factor erythrobia-2 related factor 2 (Nrf2)-antioxidant response element (ARE) pathway, which protected human endothelial cells against oxidative stress [43]. Garlic saponins were reported to protect mouse-derived C2C12 myoblasts against growth inhibition and DNA damage induced by H_2_O_2_, and to scavenge intracellular reactive oxygen species (ROS) [44]. 

In summary, garlic and its active ingredients (such as phenols and saponins) have certain antioxidant effects. Different processing methods also affected the antioxidant activity of garlic. Usually, raw garlic had a stronger antioxidant activity than cooked garlic, and the antioxidant activity of fermented garlic, such as black garlic, was stronger than that of crude garlic. In addition, the cellular experiment showed that the mechanism of antioxidative action of garlic might be involved with the enhancement of antioxidant enzyme activities and the regulation of the Nrf2-ARE pathway.

### 3.2. Anti-Inflammatory Activity

Garlic and its bioactive compounds have also been shown to exhibit anti-inflammatory properties. In a study, the ethyl linoleate in garlic reduced the production of nitric oxide (NO) and prostaglandin E-2 by down-regulating the expression of inducible NO synthase (iNOS) and cyclooxygenase-2 (COX2) in lipopolysaccharide-stimulated RAW 264.7 macrophages [45]. Another study revealed that the garlic 14-kDa protein inhibited the inflammatory mediators including NO, TNF-α, and interleukin (IL) -1β by inhibiting the transcription factor nuclear factor-kappa B (NF-κB) signaling pathway in lipopolysaccharide-stimulated J774A.1 macrophages [46]. In addition, AGE inhibited inflammation in apolipoprotein E-knockout mice. The treatment of AGE reduced the level of tumor necrosis factor-α (TNF-α) and the IL-1 receptor-associated kinase 4 and enhanced the activity of the adenosine monophosphate-activated protein kinase (AMPK) in the liver [47]. Moreover, allicin could be used as a potential complementary treatment against the inflammatory response induced by schistosome infection in BALB/c mice [48]. Furthermore, garlic supplements alleviated osteoarthritis in obese or overweight patients by reducing resistin [49].

Collectively, in both in vitro and in vivo experiments, garlic could inhibit inflammation mainly by inhibiting inflammatory mediators, such as NO, TNF-α, and IL-1. Garlic has great potential to treat inflammatory diseases, such as arthritis, in humans, because of its low or absent toxicity.

### 3.3. Antimicrobial Activity

Garlic has a broad spectrum of antibacterial and antifungal properties [50,51,52]. The antibacterial activities of two special varieties of garlic, “Rosato” and “Caposele”, from the Campania region in Italy were analyzed. It was found that the Caposele variety could significantly suppress the growth of *Aspergillus versicolor* and *Penicillum citrinum*, while the Rosato variety had a stronger inhibitory effect on *Penicillium expansum* [53]. Moreover, AGE was effective in inhibiting *Burkholderia cepacian* [54]. Garlic oil also had antibacterial activities and could restrict the growth of *Staphylococcus aureus, Escherichia coli*, and *Bacillus subtilis* [50]. It was found that garlic oil inhibited the fungus *Penicillium funiculosum*, probably by penetrating into cells and organelles, destroying the cell structure, and inducing the leakage of cytoplasm and macromolecules [55]. Additionally, garlic oil was found to disrupt the normal metabolism of *Candida albicans,* which is associated with the induction of key genes involved in oxidative phosphorylation, the cell cycle, and protein processing in the endoplasmic reticulum [56]. Furthermore, in a clinical trial, the treatment of raw garlic inhibited *Helicobacter pylori* in the stomach of patients with *H. pylori* infection [57].

In a word, the antibacterial effects of garlic are related to its varieties and processing methods. Garlic oil was demonstrated to be the main antibacterial ingredient that destroys the structure and the metabolic process of bacterial cells.

### 3.4. Modulating Immune System

Garlic contains many bioactive compounds that are beneficial for the immune system. Garlic polysaccharides have an immunomodulatory effect and regulate the expressions of IL-6, IL-10, TNF-α, and interferon-γ in RAW 264.7 macrophages. Compared with black garlic, polysaccharides in fresh garlic exhibit a more potent activity in immunomodulation. This is probably due to the degradation of fructan constituents during processing [58]. In an in vivo study, the treatment of garlic oil 30 minutes before the administration of diazinon to Wistar rats can normalize several immunological parameters of rats, such as their serum total immunoglobulin concentration and T-cell subtype CD4^+^ [59]. In addition, the combination of garlic oil and levamisole can significantly balance the T-helper 1 / T-helper 2 response in Wistar rats [60]. Moreover, selenylation modification of garlic polysaccharides significantly improves its immune-enhancing activity, and selenizing garlic polysaccharides promotes lymphocyte proliferation, enhances interferon-γ and IL-2, and increases the serum antibody titer in 14-day-old chickens [61]. Moreover, the consumption of AGE was found to reduce the occurrence and severity of the cold and flu and improve the immune system functions in humans [8]. Overall, polysaccharides appear to be the main immune-modulating components in garlic.

### 3.5. Cardiovascular Protection

Recently, the numbers of deaths from cardiovascular diseases have significantly risen [62]. There has been a growing interest in natural products to protect the cardiovascular system, and garlic is one of the most promising candidates [63,64,65,66,67]. It has been demonstrated that the intake of garlic powder can effectively reduce blood pressure, total cholesterol, low-density lipoprotein cholesterol, and other risk factors related to cardiovascular diseases [68]. 

#### 3.5.1. Antihypertensive Activity

**Figure 2:**
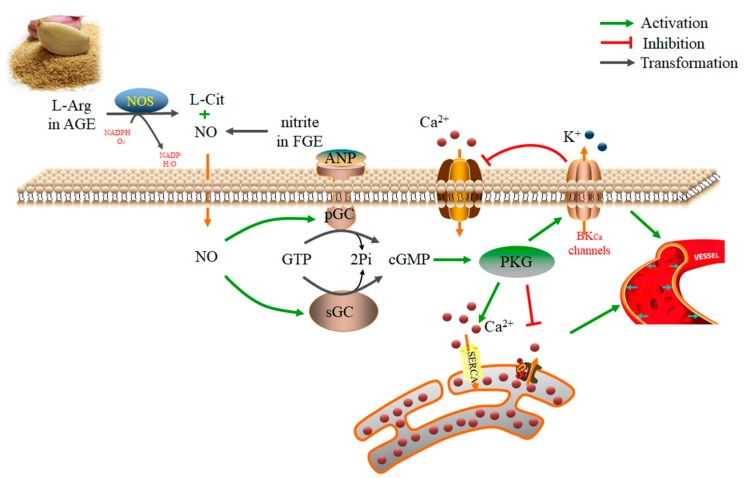
The mechanisms of the antihypertensive properties of garlic extract via increasing the production of nitric oxide (NO) in vascular smooth muscle cells. The l-arginine (L-Arg) in aged garlic extract (AGE) could be transformed into NO and L-citruline (L-Cit) mediated by nitric oxide synthase (NOS). Moreover, the nitrite in the fermented garlic extract (FGE) could be converted into NO in vivo by *Bacillus subtilis*. NO and atrial natriuretic peptide (ANP) activated particulate guanylyl cyclase (pGC) and soluble guanylyl cyclase (sGC), thus catalyzing the transform of guanosine triphosphate (GTP) to cyclic guanosine monophosphate (cGMP). The elevated cGMP activated PKG, and PKG decreased intracellular Ca^2+^ concentration by increasing intracytoplasmic Ca^2+^ transport into the sarcoplasmic reticulum through the sarco/endoplasmic reticulum Ca^2+^-ATPase (SERCA) pathway, thereby preventing the release of Ca^2+^ from the sarcoplasmic reticulum to the cytoplasm, and stimulating the Ca^2+^-activated K^+^ (BK_Ca_) channel on the cell membrane, as well as reducing the Ca^2+^ influx. As a result, the vascular smooth muscle relaxed, and the blood vessels dilated.

Garlic can also reduce oxidative stress, increase the production of NO and hydrogen sulfide (H_2_S), and inhibit the angiotensin converting enzyme, thereby lowering hypertension [69,70,71,72,73,74]. A study showed that AGE could stimulate the production of NO, leading to endothelial-dependent vasodilation in the isolated rat aortic rings. Moreover, l-arginine in AGE was crucial in the NOS-mediated NO production [71]. In addition, S-1-propylenecysteine was shown to be the key antihypertensive compound in the AGE. S-1-propylenecysteine was shown to improve peripheral blood circulation and reduce the systolic blood pressure in spontaneous hypertension rats, without affecting the systolic blood pressure of control rats [75]. In another study, the nitrites in the fermented garlic extract (FGE) could be converted into NO in vivo by *Bacillus subtilis*. Further, NO reduced the systolic blood pressure in spontaneous hypertension rats through the soluble guanylyl cyclase (sGC)-cyclic guanosine monophosphate (cGMP)-protein kinases G (PKG) pathway [76]. Also, FGE was shown to alleviate pulmonary hypertension by decreasing the expression of vascular endothelial cell adhesion molecule-1 and matrix metalloproteinase-9 (MMP-9) and increasing the expression of PKG and endothelial nitric oxide synthase (eNOS) in monocrotaline-induced pulmonary hypertension rats [77]. Moreover, the combination of garlic and its bioactive compound alliin and captopril increased the activity of captopril on inhibiting angiotensin-converting enzyme (ACE) and hypertension in rats [72]. In a placebo-controlled trial, 44 patients with hypertension were given enzymatic browning processed garlic, and their systolic blood pressure and the diastolic blood pressure were significantly reduced [78]. The anti-hypertensive mechanisms of garlic are shown in Figure 2.

#### 3.5.2. Anti-hyperlipidemic Activity

Studies demonstrate that garlic can lower blood lipids in animals and people. A study showed that high temperature and high pressure processing could remove the pungency of garlic, and this garlic effectively reduced the levels of total cholesterol, low-density lipoprotein cholesterol, and triglyceride in high-cholesterol diet-fed Sprague–Dawley rats [79]. Another study found that adding 1.5% black garlic extract in high-fat diet for male Sqrague-Dawley rats could significantly modulate the metabolism of lipids and cholesterol and decrease the total levels of blood lipids, triglyceride, and cholesterol, which could be due to the reduction of the mRNA expression of sterol regulatory element binding protein-1c [80]. In a cross sectional study, the intake of garlic (300 mg/day, 8 weeks) was shown to reduce the levels of cholesterol and low-density lipoprotein and elevate the level of high-density lipoprotein, but garlic had no effect on the level of triglycerides in patients with diabetic dyslipidemia [81]. Additionally, a supplement of aged garlic for 13 weeks was found to reduce the activities of myeloperoxidase and lipid hydroperoxide in serum and to decrease the concentrations of F_2_-isoprostanes in plasma and urine in 41 patients with hypercholesterolemia. Moreover, aged garlic had better effects than raw garlic [82].

#### 3.5.3. Heart Protection

Garlic can also protect the heart. Garlic has been shown to increase Na^+^/K^+^-ATPase protein levels and reduce cardiac hypertrophy and remodeling induced by isoproterenol in rats [83]. Another study indicated that garlic extract activated the sirtuin 3-manganese superoxide dismutase pathway by deacetylating manganese superoxide dismutase, thus protecting the heart function in streptomycin-induced diabetic rats [84]. In addition, adding garlic and fenugreek into the diet improved pathological changes of heart tissues in rats [85]. Garlic extract had protective effects on heart rate variability and improved cardiac, as well as mitochondrial, dysfunction in insulin-resistant obese rats [86]. Moreover, garlic extract treatment ameliorated the heart tissue in a rat model of gentamicin-induced chronic renal failure and induced a reduction in oxidative stress and controlled Na^+^/K^+^-ATPase activity and Ca^2+^ levels [87]. Furthermore, AGE had a dose-dependent protective effect against isoproterenol-induced cardiotoxicity. SAC combined with atenolol was more effective against isoproterenol-induced myocardial dysfunction in rats [88]. Notably, allicin was easily degraded into organic diallyl polysulfide in the presence of thiols, which was able to effectively provide H_2_S to protect the heart [13]. 

#### 3.5.4. Other Cardiovascular Protective effects 

Garlic also has other cardiovascular protective effects. Garlic was reported to inhibit platelet aggregation, which might be related to the antioxidant activity of garlic and its antioxidant compounds [13,89]. The polyphenols in aged black garlic extract had a relaxing effect on coronary arteries before and after ischemia-reperfusion (I/R) in rat hearts and improved myocardial contractility [90]. Moreover, AGE treatment inhibited inflammatory response to prevent atherosclerosis by reducing the serum level of C-reactive protein and thromboxane B-2, the protein level of TNF-α and IL-1 receptor-associated kinase 4, and increasing AMPK activity in the liver of apolipoprotein E-knockout mice [47]. Furthermore, AGE inhibited the vascular inflammation and lipid deposition in progress of atherosclerosis at an early stage in the apolipoprotein E-knockout mice, and AGE also inhibited the development of coronary artery calcification in humans [91].

In short, numerous studies have found that garlic exhibits protective cardiovascular effects and can alleviate hypertension, hyperlipidemia, and heart disease. Garlic’s mechanisms of action could be mainly related to the reduction of oxidative stress, suppression of angiotensin converting enzymes, a reduction of lipid peroxidation, and an increase of NO and H_2_S production.

### 3.6. Anticancer Activity

Cancer is acknowledged to be a primary cause of death in the world, and various natural products like berries, cruciferous vegetables, tomatoes, and ginger have been demonstrated to possess anticancer properties [92,93,94,95,96,97,98,99,100]. Recent studies have also shown that garlic and its active constituents can protect against diverse cancers, such as colorectal, lung, gastric, and bladder cancers [101,102,103,104,105,106]. 

#### 3.6.1. Regulating Metabolism of Carcinogenic Substances

People are exposed to various carcinogens in their daily lives [107]. A study revealed that garlic and its sulfur compounds can diminish the activation of carcinogens, thus reducing the risk of cancer [108]. In addition, garlic and its organic allyl sulfides can inhibit the generation of nitrosamines, a kind of carcinogen produced during cooking and storage [109,110]. Moreover, garlic allyl sulfides can block DNA alkylation, which is an early step in nitrosamine carcinogenesis [108]. 

#### 3.6.2. Suppressing Cell Growth and Proliferation

Cancer cells have the characteristic of infinite proliferation [111]. It was reported that crude garlic extract exhibited an anti-proliferative effect on human cancer cell lines, including liver (HepG2), colon (Caco2), prostate (PC-3), and breast (MCF-7) cancer cells [112]. Garlic extract induced G_2_/M-phase cell cycle arrest in EJ bladder cancer cells by activating ataxia-telangiectasia mutated and checkpoint kinase 2, and then inhibiting the phosphorylation of Cdc25C (Ser216) and Cdc2 (Thr14/Tyr15), down-regulating the expression of Cyclin B1, and up-regulating p21WAF1 [113]. The bioactive compounds of garlic, DATS, can suppress the proliferation of SGC-7901 gastric cancer cells and block the cell cycle in the G_2_/M-phase [114]. Moreover, SAC can induce G_1_/S-phase cell cycle arrest in A2780 human epithelial ovarian cancer cells [115]. S-propargyl-l-cysteine (SPRC), an analogue of SAC, reduced the proliferation of human pancreatic ductal adenocarcinoma cells and induced cell cycle arrest in the G_2_/M-phase [116]. The garlic derived S-allylmercaptocysteine (SAMC) suppressed the proliferation of hepatocellular carcinoma cells and negatively affected the cell cycle. It decreased the percentage of the S phase and increased the percentage of the G_0_/G_1_ phase [117]. Another study showed that SAMC could inhibit the proliferation of human colorectal carcinoma SW620 cells [118]. Moreover, allicin was found to inhibit the proliferation of gastric adenocarcinoma cells. It induced cell cycle arrest in the S-phase, without affecting normal intestinal cells (INT-407) [18]. Furthermore, ajoene was shown to restrain the growth of glioblastoma multiforme cancer stem cells and human breast cancer cells [119,120]. 

**Figure 3:**
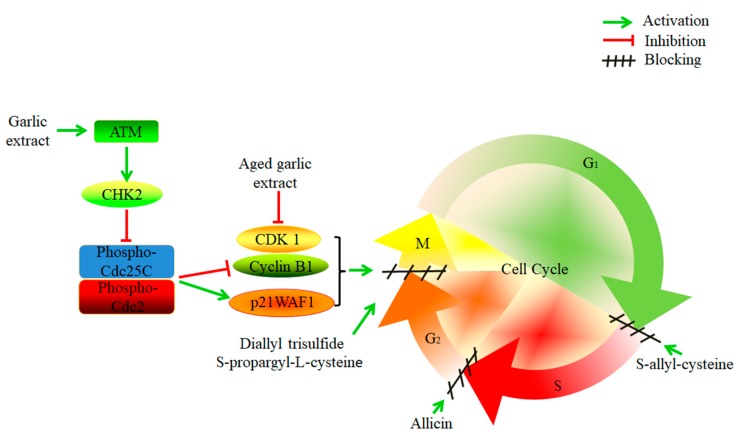
The mechanisms of garlic and its active compounds on the inhibition of the cell cycle in cancer cells. Garlic extract activated ataxia-telangiectasia mutated (ATM) and checkpoint kinase 2 (CHK2), and inhibited the phosphorylation of Cdc25C and Cdc2, which down-regulated cyclin B1 and up-regulating p21WAF1, thereby inhibiting the cell cycle in the G_2_/M-phase. Aged garlic extract can down-regulate Cyclin B1 and Cyclin-dependent kinase 1 (CDK1) and block the cell cycle in the G_2_/M-phase. Diallyl trisulfide and S-propargyl-l-cysteine can also block the cell cycle in the G_2_/M-phase. Moreover, S-allyl-cysteine induced cell cycle arrest in the G_1_/S-phase, and allicin induced cell cycle arrest during the S-phase.

In addition, AGE had anti-tumor effects on 1,2-dimethylhydrazine (DMH)-induced colon cancer in rats and could delay cell cycle progression during the G_2_/M-phase by inactivating the NF-κB signaling pathway and down-regulating the expression of Cyclin B1 and Cyclin-dependent kinase 1 [121]. Another study found that ethanol-based garlic extract suppressed the growth of multiple myeloma and prostate cancer cells in vitro. The growth of mammary tumor cells was also suppressed in vivo by ethanol-based garlic extract by increasing stress on the endoplasmic reticulum [122]. Figure 3 shows the mechanisms of cell cycle inhibition by garlic. 

#### 3.6.3. Inducing Apoptosis

The intake of raw and crushed garlic was found to upregulate apoptotic-related genes, such as aryl hydrocarbon receptor, hypoxia-inducible factor 1α, and proto-oncogene c-Jun, which influenced the expression of genes related to immunity and cancer in the blood of human beings [123]. DATS induced apoptosis via the accumulation of B cell lymphoma-2 (Bcl-2) associated X (Bax), p53 and cytochrome C, and the decrease of Bcl-2 expression in SGC-7901 gastric cancer cells. DATS also significantly induced tumor apoptosis in a mouse model with SGC-7901 gastric cancer cells [114]. In addition, SAC could induce the apoptosis of A2780 human epithelial ovarian cancer cells, decrease pro-caspase-3, poly(ADP-ribose) polymerase-1 (PARP-1) and Bcl-2, and increase the expression of active caspase-3 and Bax protein [115]. SAMC induced apoptosis via the Jun *N*-terminal kinase (JNK) and p38 mitogen activated protein kinase (p38 MAPK) pathways in SW620 human colorectal carcinoma cells [118]. Moreover, alliin was shown to induce the apoptosis of gastric adenocarcinoma cells by producing ROS and could decrease the membrane potential of mitochondria through down-upregulating the protein level of Bax/Bcl-2 and up-regulating cytochrome C [18]. Furthermore, S-propargyl-l-cysteine led to apoptosis in human pancreatic ductal adenocarcinoma cells and limited tumor growth in the Panc-1 xenograft model in vivo by activating the JNK pathway [116]. 

#### 3.6.4. Suppressing Angiogenesis

It was reported that AGE inhibited cell motility, proliferation, and tube formation of ECV304 endothelial cells and the transformed rat lung endothelial cells [124]. Further, DATS effectively inhibit the angiogenesis in MDA-MB-231 human breast cancer cells [125]. Furthermore, the combination of garlic and lemon aqueous extracts had an inhibitory effect on EMT6/P breast cancer cells in BALB/c mice by inhibiting the expression of vascular endothelial growth factor (VEGF) and, finally, angiogenesis [126].

#### 3.6.5. Inhibiting Invasion and Migration 

Both invasion and migration are malignant behaviors of tumor cells [111]. Garlic extract inhibited the expression of MMP-9, reduced the binding activity of the transcription factor activator protein 1 (AP-1) (specificity the protein-1 and NF-κB motifs), and increased the expression of heat shock protein A6, which blocked the migration and invasion of bladder cancer EJ cells [113]. AGE decreased the invasive capacity of SW480 and SW620 colorectal cancer cells by inhibiting cell motility and cell proliferation [124]. In addition, SAC reduced the migration of A2780 epithelial ovarian cancer cells by significantly reducing the expression of wingless-type MMTV integration site family member 5A (Wnt5a), phosphor-protein kinase B, and c-Jun proteins [115]. In another study, DATS inhibited SGC-7901 gastric tumor cell migration and invasion by regulating the protein expressions of MMP-9 and E-cadherin in BALB/c-nude mice [114].

#### 3.6.6. Alleviating the Adverse Effects of Anticancer Therapies

Garlic has been indicated to mitigate the adverse effects of several anticancer therapies [127]. In one study, AGE improved the renal histological, ultrastructural, and biochemical changes induced by cisplatin therapy, such as hemorrhaging, glomerular atrophy, tubular necrosis, and degeneration in adult male rats [128]. Furthermore, allicin enhanced the anticancer effect of tamoxifen in mice and reduce the liver injury caused by tamoxifen treatment. Allicin improved the tamoxifen-induced changes in the levels of superoxide dismutase, glutathione, aspartate aminotransferase, alkaline phosphatase, and alanine aminotransferase [129]. Additionally, garlic had a protective effect against febrile neutropenia in patients who received chemotherapy for hematological malignancies in the lower-risk febrile neutropenia subgroup [130].

#### 3.6.7. Other Anti-Cancer Actions

AGE was shown to enhance the in vitro production of interferon-γ in splenocytes and increase the ratio of CD4^+^/CD8^+^ on implanted fibrosarcoma tumors in BALB/c mice, which improved the immune responses of mice to fibrosarcoma and inhibited tumor growth [131]. Additionally, the garlic and lemon aqueous extract were demonstrated to activate the immune system against implanted breast cancer in mice by increasing interferon-γ, IL-2, and IL-4 levels [126]. Moreover, DADS prevented colorectal tumorigenesis induced by azoxymethane and dextran sulfate in FVB/N mice. The treatment of DADS reduced inflammation by suppressing glycogen-synthase kinase-3β and the nuclear localization of NF-κB [132].

In summary, garlic and its active components can prevent and manage different cancers (Table 1). These anticancer mechanisms include the regulation of carcinogen metabolism, inhibition of cell growth and proliferation, induction of apoptosis, suppression of angiogenesis, and inhibition of invasion and migration. Garlic can also diminish the negative effects of anticancer therapies.

### 3.7. Hepatoprotective Activity

Accumulating studies have revealed that several natural products, including garlic, had hepatoprotective effects [133,134,135]. In an in vitro study, the black garlic extract reduced the damage of tert-butyl hydroperoxide in rat clone-9 hepatocytes by inhibiting apoptosis, lipid peroxidation, oxidative stress, and inflammation [136]. In another study, garlic attenuated liver damage induced by alloxan in rats and improved the biochemical plasma factors of hepatic functions, such as urea, creatinine, aspartate transaminase, and alanine transaminase [137]. In addition, the combination of garlic and ascorbic acid protected against the liver toxicity induced by Cd in albino mice [138]. Moreover, single clove garlic had a stronger protective effect than multi-clove garlic on CCl_4_-induced acute liver injury in male rabbits [38].

Moreover, garlic oil was shown to enhance the activities of hepatic antioxidant enzymes, block metabolic activation of 1,3-dichloro-2-propanol, and reduce apoptosis in the liver, indicating a protective effect against liver injury in rats [139]. Also, it was reported that the active compounds of garlic, such as DAS, DADS, and S-methyl-l-cysteine, could prevent and treat liver damage, such as acute and chronic ethanol-induced liver damage [140]. DADS in garlic essential oil was found to attenuate nonalcoholic fatty liver disease, which was induced by a high-fat diet in rats. DADS significantly reduced the release of pro-inflammatory cytokines in the liver and increased antioxidant activity by inhibiting the expression of cytochrome P450 2E1 [141]. 

Compared with unfermented garlic extract, the fermented garlic extract by *Lactobacillus plantarum* BL2 (LAFGE) was able to more effectively reduce liver lipid levels and ameliorate hepatic steatosis in mice [142]. Another study revealed that LAFGE inhibited liver cell apoptosis partly by suppressing MAPK phosphorylation and down-regulating p53, which protected the liver from acetaminophen-induced injury in rats [143]. Furthermore, LAFGE was considered as a potential treatment for mild hepatic dysfunction. In a double-blind, randomized, placebo-controlled study, 36 adults with mildly high level of serum gamamyl glutamyl transpeptiase (GGT) received LAFGE, and the levels of GGT and alanine aminotransferase were improved without adverse effects [144]. However, an animal study showed that an overdose of garlic has negative morphological effects on the liver. After 30 days of injecting fresh garlic extract at 500 mg and 1000 mg/kg via a gastric tube in Wistar albinism rats, bleeding and nodular swelling appeared on the external surfaces of the liver, and the weight of the liver increased [145]. 

Generally speaking, garlic can effectively alleviate acute or chronic liver injury, but the side effects of excessive consumption of garlic also need to be considered. It is necessary to evaluate the safe dose and duration of garlic usage in humans.

### 3.8. Digestive System Protection

Garlic has been reported to have therapeutic efficacy against gastric tissue injury. A study showed that black garlic extract can stimulate gastrointestinal peristalsis, promote gastrointestinal emptying, and facilitate defecation. The water fraction of black garlic had a better effect on improving gastrointestinal functions compared with the *n*-butanol fraction and ethyl acetate fraction in the small intestine in vitro [146]. Additionally, the treatment by garlic and cabbage extract reduced the length of gastric ulcers, total gastric acid, gastric juice volume, total bacteria count, and changes in histopathology. This treatment also improved the pH value of gastric juice in rats [147]. Furthermore, the oral administration of AGE was shown to heal gastric mucosal injury induced by indomethacin in male rats and reduce the total microbial amount in the stomach [148]. AGE was effective in preventing indomethacin-induced ulcers in rats via the reduction in oxidative stress and the elevation of prostaglandin E-2, glutathione, and NO in gastric tissue [149]. 

The bioactive compounds in garlic were also crucial in the protection of the digestive system. Allicin was demonstrated to inhibit the activation of the AP-1/NF-κB/signal transducer and activator of transcription-1 (STAT-1) by inhibiting the phosphorylation of p38, JNK, and the extracellular signal-regulated kinase 1/2 (ERK1/2)-regulated peroxisome proliferator-activated receptor (PPAR)-γ, which alleviated ulcerative colitis in mice [150]. DADS reduced interferon-inducible protein-10, IL-6, and DAS inhibited NO, as well as the expression of STAT-1 in interferon-γ-stimulated intestinal cells. Further, DAS and DADS improved the colitis induced by dinitrobenzenesulfonic acid in mice [70]. In addition, intake of raw garlic was shown to decrease bacterial urease activity and reduce *Helicobacter pylori* in the stomach of 15 patients [57]. 

In general, garlic and its bioactive compounds can improve gastrointestinal functions and alleviate colitis, gastric ulcers, and other gastrointestinal diseases by reducing oxidative stress, inhibiting inflammation, and decreasing *Helicobacter pylori*.

### 3.9. Anti-Diabetic Activity

Garlic has been shown to reduce pancreatic cell injury, oxidative stress, and pathological changes in streptomycin-induced type 1 diabetic rats [151]. In addition, garlic had a protective effect on diabetic retinopathy in diabetic rats. The weight, blood glucose, and morphological changes of retinal tissue in the group treated with garlic improved after 7 weeks of gastric gavage of raw garlic extract in rats [152]. Moreover, AGE had a dose-dependent anti-diabetic effect on streptomycin-induced diabetic rats [153]. Furthermore, a meta-analysis was performed on 768 patients with type 2 diabetes mellitus in nine randomized controlled trials, and the result showed that garlic supplements significantly reduced fructosamine and glycosylated hemoglobin. This study demonstrated that garlic supplements were effective in the management of type 2 diabetes mellitus [154]. Thus, garlic and its bioactive components might be effective agents to help treat diabetes and diabetic complications.

### 3.10. Anti-Obesity Activity

Garlic oil has anti-obesity properties and has been shown to counteract the influence of a high-fat diet on the weight of body and adipose tissue in hyperlipidemia rats [155]. In addition, the oral administration of LAFGE reduced the weight of high-fat diet male C57BL/6J mice. LAFGE also reduced their epididymal, retroperitoneal, and mesenteric adipose tissue mass. The possible mechanism of action was that LAFGE inhibited lipogenesis by down-regulating the mRNA and protein expression of PPAR-γ, C/EBPα, and lipogenic proteins [9]. Moreover, the methanolic extract of black garlic was found to reduce the weight of rats fed with a high-fat diet. This treatment regulated lipid metabolism by up-regulating the expression of AMPK, forkhead box protein O1, perilipin, and adiponectin in the adipose tissue of the rats and down-regulating the cluster of differentiation 36 (CD36), plasminogen activator inhibitor 1, resistin, and TNF-α [156]. 

Collectively, the studies demonstrate that fermented garlic products have certain positive effects on obesity by inhibiting lipogenesis and regulating lipid metabolism.

### 3.11. Neuroprotection

A study revealed that AGE and its carbohydrate derivative N-α-(1-deoxy-D-fructos-1-yl)- l-arginine alleviated neuroinflammation by inhibiting the production of NO and regulating the expression of multiple protein targets related to oxidative stress in lipopolysaccharide-activated murine BV-2 microglial cells [157]. In another study, the anti-neuritis activity of garlic was related to the organosulfur compounds in lipopolysaccharide-stimulated BV2 microglia cells [158]. The treatment of garlic during pregnancy and lactation was shown to decrease the concentration of Pb in the blood and brain and partially prevent the Pb-induced apoptosis of neurons during the hippocampal development in rats [159]. The Basso, Beattie, and Bresnahan (BBB) scoring system was used to conduct a neurological evaluation on the influence of AGE on a spinal cord I/R model of rats. Compared with the I/R group, the BBB score of the AGE group was markedly higher, thus demonstrating that AGE had a significant neuroprotective effect [160]. Additionally, AGE attenuated the loss of cholinergic neurons and enhanced the level of vesicular glutamate transporter 1 and glutamate decarboxylase in the hippocampal area of rats, which attenuated the damage of working memory [161]. Moreover, the ethanol extract of garlic was shown to improve memory. Garlic activated Na^+^/K^+^ ATPase, Ca^2+^ ATPase, and glutamine synthetase in the hippocampus of diabetic Wistar rats [162]. Furthermore, the ethanol extract of fermented garlic effectively prevented working memory via the damage induced by monosodium glutamate [163]. Z-ajoene was able to prevent I/R-induced delayed neuronal death and gliosis and reduce the lipid peroxidation in the CA1 region of the hippocampus [15]. Moreover, SAC ameliorated cognitive impairment in rats by reducing oxidative stress, neuroinflammation, astrogliosis, and acetylcholinesterase activity [164].

In conclusion, both in vivo and in vitro experiments showed that garlic has significant neuroprotective properties and mainly acts on the hippocampus. Organic sulfur compounds were shown to play a major role in neuroprotection.

### 3.12. Renal Protection

Garlic was shown to effectively alleviate nephrotoxicity [10]. The aqueous extract of garlic was shown to reduce the oxidative stress in the kidneys of diabetic rats [165]. In addition, the aqueous extract of garlic improved the renal plasma biochemical factors induced by alloxan in Wistar rats [137]. Moreover, DATS was reported to activate the Nrf2-ARE pathway, protecting the kidney from oxidative stress injury induced by arsenic in rats [166].

## 4. Conclusions

Garlic is a widely consumed spice with a characteristic odor. It contains many bioactive components, such as organic sulfides, saponins, phenolic compounds, and polysaccharides. The organic sulfides, such as allicin, alliin, diallyl sulfide, diallyl disulfide, diallyl trisulfide, ajoene, and S-allyl-cysteine, are major bioactive components in garlic. Garlic and its bioactive components show many biological functions, such as antioxidant, anti-inflammatory, immunomodulatory, cardiovascular protective, anticancer, hepatoprotective, digestive system protective, anti-diabetic, anti-obesity, neuroprotective, renal protective, antibacterial, and antifungal activities. Generally, garlic is non-toxic or has low toxicity. Therefore, garlic and its bioactive compounds are promising as functional foods or nutraceuticals for the prevention and treatment of different diseases. In the future, more biological functions of garlic should be evaluated, and the relative compounds of garlic need to be separated and identified. More investigations should be conducted to deeply illustrate garlic’s mechanisms of action. In addition, the effects of the processing, such as fermentation and heat, on garlic should be further studied because they could impact the biological functions and safety of garlic. Furthermore, more clinic trials should be carried out to confirm the health benefits of garlic on humans, and special attention should be paid to the side effects/safety of garlic.

## Figures and Tables

**Table 1:** The biological activities of garlic and its active components.

Product	Compounds	Study Type	Subjects/ Cell Lines	Main Effects	Possible Mechanisms	Ref.
**Antioxidant Activity**						
Aged garlic extract		In vitro	Human endothelial cells	Protecting cells against oxidative stress	Inducing the expression of several antioxidant enzymes, HO-1 and GCLM subunit, through Nrf2- ARE pathway	[43]
	Saponins	In vitro	Mouse-derived C2C12 myoblasts	Protecting cells against the growth inhibition and DNA damage induced by H_2_O_2_	Scavenging intracellular reactive oxygen species	[44]
**Anti-inflammatory Activity**						
	Ethyl linoleate	In vitro	Lipopolysaccharide-stimulated RAW 264.7 macrophages	Reducing the production of NO and prostaglandin E-2	Down regulating the expression of iNOS and COX2	[45]
	Garlic 14-kDa protein	In vitro	Lipopolysaccharide-stimulated J774A.1 macrophages	Inhibiting the inflammatory mediators such as NO, TNF-α, and IL-1β	Inhibiting the transcription factor NF-κB signaling pathway	[46]
Aged garlic extract		In vivo	Apolipoprotein E-knockout mice	Inhibiting inflammation	Reducing the level of TNF-α and interleukin IL-1 receptor-associated kinase 4 Increasing the activity of AMPK in the liver	[47]
	Allicin	In vivo	BALB/c mice	Protecting against the inflammatory response induced by schistosome infection		[48]
Garlic tablets (equal to 2.5 g fresh garlic daily)		Clinical trial	40 post-menopausal obese or overweight patients	Alleviating osteoarthritis	Reducing resistin	[49]
**Antimicrobial Activity**						
Garlic “Rosato” and “Caposele”		In vitro	*Aspergillus versicolor*, *Penicillum citrinum* and *Penicillium expansum*	Inhibiting the growth of bacteria		[53]
Aged garlic extract	Allicin	In vitro	*Burkholderia cepacian*	Inhibiting the growth of bacteria		[54]
	Garlic oil	In vitro	*Staphylococcus aureus, Escherichia coli* and *Bacillus subtilis*	Inhibiting the growth of bacteria		[50]
		In vitro	*Penicillium funiculosum*	Inhibiting the growth of bacteria	Penetrating into cells and organelles Destroying the cell structure Leading to the leakage of cytoplasm and macromolecules	[55]
		In vitro	*Candida albicans*	Disrupting the normal metabolism of bacteria	Inducing key genes involved in oxidative phosphorylation, the cell cycle, and protein processing in the endoplasmic reticulum	[56]
Raw garlic		Clinical trial	15 patients with *H. pylori* infection	Inhibiting *Helicobacter pylori* in the stomach		[57]
**Modulating Immune System**						
Fresh garlic	Polysaccharides/ Fructan	In vitro	RAW 264.7 macrophages	Exerting immunomodulatory effect	Regulating the expressions of IL-6, IL-10, TNF-α, and interferon-γ	[58]
	Garlic oil	In vivo	Wistar rats	Normalizing several immunological parameters of rats, such as the serum total immunoglobulin concentration and T-cell subtype CD4^+^ Combination of garlic oil and levamisole could balance the T-helper 1/ T-helper 2 response		[59,60]
	Selenizing garlic polysaccharides	In vivo	14-day-old chickens	Promoting lymphocyte proliferation Enhancing interferon-γ and IL-2 Increase the serum antibody titer		[61]
Aged garlic extract		Clinical trial	56 healthy human participants	Reducing the occurrence and severity of cold and flu Improving the immune system functions		[8]
**Cardiovascular Protection**						
Aged garlic extract		In vitro	Isolated rat aortic rings	Leading to endothelial-dependent vasodilation	Stimulating the production of NO	[71]
Aged black garlic extract	Polyphenols	In vivo	Isolated hearts of male Sprague–Dawley rats	Relaxing coronary arteries before and after ischemia-reperfusion in rat Preventing the decrease of myocardial contractility		[90]
Aged garlic extract	S-1-propylenecysteine	In vivo	Spontaneous hypertension rats	Improving peripheral blood circulation Reducing the systolic blood pressure		[75]
Fermented garlic extract by *Bacillus subtilis*		In vivo	Spontaneous hypertension rats	Reducing the systolic blood pressure	Modulating the sGC-cGMP-PKG pathway	[76]
Fermented garlic extract		In vivo	Monocrotaline-induced pulmonary hypertension rats	Alleviating pulmonary hypertension	Decreasing the expression of vascular endothelial cell adhesion molecule-1 and MMP- 9Increasing the expression of PKG and eNOS	[77]
Garlic	Alliin	In vivo	Female Wistar albino rats	Increasing the activity of captopril on inhibiting ACE and hypertension		[72]
1.5% black garlic extract		In vivo	High-fat diet-fed male Sprague–Dawley rats	Modulating the metabolism of lipid and cholesterol Decreasing the levels of blood total lipids, triglyceride, and cholesterol	Reducing the mRNA expression of sterol regulatory element binding protein-1c	[80]
Raw garlic	Allyl methyl sulfide/Allyl methyl sulfoxide	In vivo	Male Sprague–Dawley rats	Reduce cardiac hypertrophy remodeling induced by isoproterenol	Increasing Na^+^/K^+^-ATPase protein level	[83]
Raw garlic		In vivo	Streptomycin-induced diabetic rats	Protecting the heart function Activating sirtuin 3-manganese superoxide dismutase pathway	Deacetylating manganese superoxide dismutase	[84]
Garlic extract		In vivo	Insulin-resistant obese rats	Protecting heart rate variability, cardiac dysfunction, and mitochondrial dysfunction		[86]
		In vivo	Rat model of gentamicin-induced chronic renal failure	Protecting the heart tissue	Reducing oxidative stress Controlling Na^+^/K^+^-ATPase activity and Ca^2+^ levels	[87]
Aged garlic extract	SAC	In vivo	Rats with myocardial dysfunction induced by isoproterenol	Protecting against cardiotoxicity		[88]
Aged garlic extract		In vivo	Apolipoprotein E-knockout mice	Inhibiting inflammatory response to prevent atherosclerosis	Reducing serum level of C-reactive protein and thromboxane B-2, protein level of TNF-α and IL-1 receptor-associated kinase 4 Increasing AMPK activity in the liver	[47]
		In vivo	Apolipoprotein E-knockout mice	Inhibiting the vascular inflammation and lipid deposition in the early stage of atherosclerosis development		[91]
High temperature and high pressure-processed garlic		In vivo	High-cholesterol diet-fed Sprague–Dawley rats	Reducing the levels of total cholesterol, low-density lipoprotein cholesterol and triglyceride		[79]
Garlic		Cohort study	30 patients with diabetic dyslipidemia	Decreasing the level of cholesterol and low-density lipoprotein Increase the level of high-density lipoprotein		[81]
Aged garlic		Clinical trial	41 patients with hypercholesterolemia	Reducing the activity of myeloperoxidase and lipid hydroperoxide in serum Decreasing the concentration of F2-isoprostanes in plasma and urine		[82]
Enzymatic browning processed garlic		Clinical trial	44 patients with hypertension	Reducing systolic blood pressure and diastolic blood pressure		[78]
**Anticancer Activity**						
Garlic extract		In vitro	Bladder cancer EJ cells	Inducing G_2_/M-phase cell cycle arrest Inhibiting cell growth Inhibiting cell migration and invasion	Activating the ATM pathway and CHK2; Inhibiting the expression of MMP-9 Reducing the binding activity of transcription factors AP-1, specificity protein-1 and NF-κB motifs Increasing the expression of heat shock protein A6	[113]
Aged garlic extract		In vitro	Colorectal cancer cell lines (SW480 and SW620) ECV304 cells and the transformed rat lung endothelial cells	Decreasing invasive activity Inhibiting cell proliferation Decreasing invasive activity Inhibiting the tube formation of endothelial cells	Inhibiting cell motility	[124]
		In vitro	DLD-1 human colon cancer cells (ATCC CCL-221)	Inhibiting cell proliferation	Down regulating the expression of cyclin B1 and CDK1 Inhibiting of activation of NF-κB	[121]
Crude garlic extract	Lipid bioactive compounds	In vitro	Human liver cancer (Hep-G2) Colon cancer (Caco-2) Prostate cancer (PC-3) Breast cancer (MCF-7) Mouse macrophage cell (TIB-71) lines	Inhibiting the growth rate of Hep-G2, PC-3, MCF-7, and TIB-71 cells by 80%–90% at 72 h (P < 0.05).	Inhibiting cell proliferation Inducing cell cycle arrest Inducing apoptosis	[112]
	Allicin	In vitro	Human gastric adenocarcinoma cell line	Inhibiting cell proliferation	Inducing cell cycle arrest at S-phase	[18]
	DATS	In vitro	Human gastric carcinoma cell line (SGC-7901)	Inhibiting cell proliferation Blocking cell cycle Increasing apoptotic cell death	Accumulating Bax, p53, and cytochrome C and decreasing the expression of Bcl-2	[114]
		In vitro	Human breast cancer cell line (MDA-MB-231)	Inhibiting angiogenesis		[125]
	Z-ajoene	In vitro	Glioblastoma multiforme cells	Inhibiting the growth of the cancer stem cells population		[119]
		In vitro	Human breast cancer cells (MDA-MB-231)	Inhibiting cell growth Inducing cell apoptosis	Targeting the folding of proteins in the endoplasmic reticulum of cancer cells	[120]
	SAC	In vitro	Human epithelial ovarian cancer cell line (A2780)	Inhibiting cell proliferation Inducing G_1_/S-phase cell cycle arrest Increasing apoptosis Reducing the migration of cells	Decreasing the expression of pro-caspase-3, Parp-1 Bcl-2 and increasing active caspase-3 and Bax Reducing the expression of Wnt5a, phosphorylation protein kinase B and c-Jun proteins	[115]
	SPRC	In vitro	Human pancreatic ductal adenocarcinoma cells (Panc-1)	Inhibiting cell proliferation Inducing apoptosis	Inducing G_2_/M-phase cell cycle arrest Regulating the level of JNK protein	[116]
	SAMC	In vitro	Human colorectal carcinoma cell line (SW620)	Inhibiting cell proliferation Inducing cell apoptosis	Regulating JNK and p38 MAPK pathways	[118]
		In vitro	Hepatoma cell lines (Hep3B and Huh-7)	Reducing the cell viability Shortening the S phase and increasing the G_0_/G_1_ phase		[117]
	Alliin	In vitro	Gastric adenocarcinoma cells	Regulating cell apoptosis	Generating reactive oxygen species Decreasing mitochondrial membrane potential by Bax/Bcl-2 Up-regulating cytochrome C	[18]
Aged garlic extract		In vivo	Adult male Wister albino rats treated with cisplatin	Improving the renal histological, ultrastructural and biochemical changes, such as hemorrhage, glomerular atrophy, tubular necrosis and degeneration		[128]
		In vivo	Fibrosarcoma tumors implanted BALB/c mice	Improving the immune responses of mice to fibrosarcoma Inhibiting tumor growth	Increasing the ratio of CD_4_^+^/CD_8_^+^ Producing interferon-γ in splenocytes	[131]
Garlic and lemon aqueous extract		In vivo	BALB/c mice xenograft model of breast cancer EMT6/P cells	Reducing tumor size Inhibiting angiogenesis Inducing apoptosis Activating the immune system	Inhibiting the expression of vascular endothelial growth factor Increasing interferon-γ, IL-2, and IL-4 levels	[126]
	Allicin	In vivo	Female Swiss albino mice	Alleviating liver injury induced by tamoxifen	Changing the decrease of superoxide dismutase, glutathione and total protein and the increase of aspartate aminotransferase, alkaline phosphatase and alanine aminotransferase levels	[129]
	DADS	In vivo	FVB/N mice	Preventing colorectal tumorigenesis induced by azoxymethane and dextran sulfate	Inhibiting inflammation Inhibiting glycogen-synthase kinase-3β Reducing the nuclear localization of NF-κB	[132]
	DATS	In vivo	Female BALB/c-nude mouse xenograft model of human gastric carcinoma SGC-7901 cells	Inhibiting tumor growth Promoting tumor apoptosis	Regulating the expressions of MMP-9 and E-cadherin protein	[114]
	SPRC	In vivo	Xenograft model of pancreatic ductal adenocarcinoma Panc-1 cells	Inhibiting tumor growth	Regulating the level of JNK protein	[116]
	SAMC	In vivo	Mouse xenograft model of hepatoma Huh-7 cells	Inhibiting tumor growth	Interacting with the Wnt-pathway co-receptor LRP6 on the cell membrane	[117]
Raw, crushed garlic		Cohort study	17 volunteers from Beltsville, Maryland	Up-regulating seven genes including *AHR, ARNT, HIF1A, JUN, NFAM1, OSM* and *REL*		[123]
Garlic extract		Cohort study	Patients who received chemotherapy for hematological malignancies	Protective effect on febrile neutropenia in lower-risk subgroup		[130]
**Hepatoprotective Activity**						
Black garlic extract		In vitro	Rat clone-9 hepatocytes	Inhibiting apoptosis, lipid peroxidation, oxidative stress, and inflammation		[136]
Garlic extract		In vivo	Wistar rats	Attenuating the liver damage induced by alloxan Improving plasma biochemical factors of hepatic function, such as urea, creatinine, aspartate transaminase, and alanine transaminase		[137]
Single clove garlic extract		In vivo	Male rabbits	Protecting against CCl_4_-induced acute liver injury		[38]
LAFGE		In vivo	C57/BL6 J mice	Reducing the liver lipid level Ameliorating the hepatic steatosis		[142]
		In vivo	rats	Inhibiting liver cell apoptosis Protecting liver from acetaminophen-induced liver injury	Suppressing MAPK phosphorylation Down regulating p53	[143]
	Garlic oil	In vivo	1,3-Dichloro-2-propanol-treated rats	Protecting liver	Enhancing the activities of hepatic antioxidant enzymes Blocking metabolic activation of 1, 3-dichloro-2-propanol Reducing the apoptosis in liver	[139]
	DADS	In vivo	Wistar rats	Protecting mice from nonalcoholic fatty liver disease induced by long-term high-fat diet.	Reducing the release of pro-inflammatory cytokines in the liver Increasing antioxidant activity by inhibiting the expression of cytochrome P450 2E1	[141]
	LAFGE	Clinical trial	36 adults with mildly high level of serum gamamyl glutamyl transpeptiase	Improving the levels of gamamyl glutamyl transpeptias and alanine aminotransferase without adverse effects		[144]
**Digestive System Protection**						
Black garlic extract		In vitro	Small intestine	Stimulating gastrointestinal peristalsis Promoting gastrointestinal emptying and facilitates defecation.		[146]
	DADS DAS	In vitro	Interferon-γ-stimulated intestinal cells	Reducing interferon-inducible protein-10, IL-6 Inhibiting NO and the expression of STAT-1		[70]
		In vivo	Male ICR mice	Improving the colitis induced by dinitrobenzenesulfonic acid		[70]
Garlic and cabbage extract		In vivo	Sprague–Dawley rats	Reducing the length of gastric ulcer, the total gastric acid, gastric juice volume, total bacteria count, and histopathological changes caused by aspirin Improving the pH value of gastric juice		[147]
Aged garlic extract		In vivo	Male albino rats	Healing the gastric mucosal injury induced by indomethacin Reducing the total microbial amount in stomach		[148]
		In vivo	Male Wistar rats	Preventing the indomethacin-induced ulcer	Reducing oxidative stress Elevating the level of prostaglandin E-2, glutathione, and NO in gastric tissue	[149]
	Allicin	In vivo	Dextran sulfate sodium-induced colitis mice	Alleviating the ulcerative colitis induced by dextran sulfate sodium	Inhibiting the activation of AP-1/NF-κB/signal transducer and activator of transcription-1 Inhibiting the phosphorylation of p38, JNK, and extracellular signal-regulated kinase 1/2 -regulated PPAR-γ	[150]
Raw garlic		Clinical trial	15 patients with *H. pylori* infection	Decreasing the bacterial urease activity Reducing the residing of *Helicobacter pylori* in the stomach		[57]
**Anti-Diabetic Activity**						
Garlic		In vivo	Diabetic rats	Protecting against diabetic retinopathy Improving weight, blood glucose, and morphological changes of retinal tissue		[152]
		Clinical trial	768 patients with type 2 diabetes mellitus	Reducing fructosamine and glycosylated hemoglobin		[154]
**Anti-Obesity Activity**						
	LAFGE	In vivo	High-fat diet-fed male C57BL/6J mice	Reducing the weight Reducing the epididymal, retroperitoneal, and mesenteric adipose tissue mass	Inhibiting the lipogenesis by down-regulating the mRNA and protein expression of PPAR-γ, C/EBPα, and lipogenic proteins	[9]
Methanolic extract of black garlic		In vivo	High-fat diet-fed male Wistar rats	Reducing the weight Regulating lipid metabolism	Upregulating the expression of AMPK, forkhead box protein O1, perilipin, and adiponectin in the adipose tissue Down-regulating cluster of differentiation 36, plasminogen activator inhibitor 1, resistin, and TNF-α	[156]
	Garlic oil	In vivo	High-fat diet-fed male Sprague–Dawley rats	Counteracting the influence of high-fat diet on the body weight and adipose tissue weight		[155]
**Neuroprotection**						
Aged garlic extract	FruArg	In vitro	Lipopolysaccharide-activated murine BV-2 microglial cells	Alleviating neuroinflammation	Inhibiting the production of NO Regulating the expression of multiple protein targets related to oxidative stress	[157]
Garlic extract		In vivo	Female Wistar rats	Reducing the concentration of Pb in the blood and brain Preventing the Pb-induced apoptosis of neurons		[159]
Aged garlic extract		In vivo	Adult male Wistar rats	Attenuating the damage of working memory	Improving the loss of cholinergic neurons Increasing the level of vesicular glutamate transporter 1 and glutamate decarboxylase in the hippocampal area	[161]
Ethanol extract of garlic		In vivo	Diabetic Wistar rats	Improving memory	Increasing the activity of Na^+^/K^+^ ATPase, Ca^2+^ ATPase, and glutamine synthetase in the hippocampus	[162]
	Z-ajoene	In vivo	Male gerbils	Preventing I/R-induced delayed neuronal death and gliosis region of the hippocampus	Reducing lipid peroxidation in the CA1	[15]
	SAC	In vivo	Male albino Wistar rats	Ameliorating the cognitive impairment	Reducing oxidative stress, neuroinflammation, astrogliosis, and acetylcholinesterase activity	[164]
**Renal Protection**						
Aqueous extract of garlic		In vivo	Type 1 diabetic rats	Reducing the oxidative stress in the kidneys		[165]
		In vivo	Wistar rats	Improving the renal plasma biochemical factors induced by alloxan		[137]
	DATS	In vivo	Male albino rats	Protecting the kidney from oxidative stress injury induced by As	Activating the Nrf2-ARE pathway	[166]

Abbreviations: ACE, angiotensin-converting enzyme; *AHR*, aryl hydrocarbon receptor; allicin, diallyl thiosulfonate; alliin, S-allyl-cysteine sulfoxide; AMPK, adenosine monophosphate-activated protein kinase; AP-1, activator protein 1; ARE, antioxidant response element; *ARNT*, aryl hydrocarbon receptor nuclear translocator; ATM, ataxia-telangiectasia mutated; Bax, Bcl-2 associated X; Bcl-2, B cell lymphoma-2; CDK1, cyclin-dependent kinase 1; cGMP, cyclic guanosine monophosphate; CHK2, checkpoint kinase 2; COX2, cyclooxygenase-2; DADS, diallyl disulfide; DAS, diallyl sulfide; DATS, diallyl trisulfide; eNOS, endothelial nitric oxide synthase; FruArg, N-α-(1-deoxy-D-fructos-1-yl)-l-arginine; GCLM, glutamate-cysteine ligase modifier; *HIF1A*, hypoxia-inducible factor 1α; HO-1, heme oxygenase-1; IL, interleukin; iNOS, inducible NO synthase; JNK, Jun *N*-terminal kinase; *JUN*, proto-oncogene c-Jun; LAFGE, fermented garlic extract by *Lactobacillus plantarum* BL2; MMP-9, matrix metalloproteinase-9; *NFAM1*, nuclear factor of activated T cells (NFAT) activating protein with immunoreceptor tyrosine-based activation motif 1; NF-κB, nuclear factor-kappa B; NO, nitric oxide; Nrf2, nuclear factor erythrobia-2 related factor 2; *OSM*, oncostatin M; Parp-1, poly (ADP-ribose) polymerase-1; PKG, protein kinases G; PPAR-γ, peroxisome proliferator-activated receptor-γ; p38 MAPK, p38 mitogen activated protein kinase; *REL*, V-relavian reticuloendotheliosis viral oncogene homolog; SAC, S-allyl-cysteine; sGC, soluble guanylyl cyclase; SAMC, S-allylmercaptocysteine; SPRC, S-propargyl-l-cysteine; STAT-1, signal transducer and activator of transcription-1; TNF-α, tumor necrosis factor-α; Wnt5a, wingless-type MMTV integration site family member 5A.
